# MicroRNA-124 Promotes Singapore Grouper Iridovirus Replication and Negatively Regulates Innate Immune Response

**DOI:** 10.3389/fimmu.2021.767813

**Published:** 2021-11-10

**Authors:** Pin-Hong Li, Li-Qun Wang, Jia-Yang He, Xiang-Long Zhu, Wei Huang, Shao-Wen Wang, Qi-Wei Qin, Hong-Yan Sun

**Affiliations:** ^1^ University Joint Laboratory of Guangdong Province, Hong Kong and Macao Region on Marine Bioresource Conservation and Exploitation, College of Marine Sciences, South China Agricultural University, Guangzhou, China; ^2^ Southern Marine Science and Engineering Guangdong Laboratory, Zhuhai, China; ^3^ Laboratory for Marine Biology and Biotechnology, Qingdao National Laboratory for Marine Science and Technology, Qingdao, China

**Keywords:** miR-124, *Epinephelus coioides*, SGIV, viral replication, immune response

## Abstract

Viral infections seriously affect the health of organisms including humans. Now, more and more researchers believe that microRNAs (miRNAs), one of the members of the non-coding RNA family, play significant roles in cell biological function, disease occurrence, and immunotherapy. However, the roles of miRNAs in virus infection (entry and replication) and cellular immune response remain poorly understood, especially in low vertebrate fish. In this study, based on the established virus-cell infection model, Singapore grouper iridovirus (SGIV)-infected cells were used to explore the roles of miR-124 of *Epinephelus coioides*, an economically mariculture fish in southern China and Southeast Asia, in viral infection and host immune responses. The expression level of *E. coioides* miR-124 was significantly upregulated after SGIV infection; miR-124 cannot significantly affect the entry of SGIV, but the upregulated miR-124 could significantly promote the SGIV-induced cytopathic effects (CPEs), the viral titer, and the expressions of viral genes. The target genes of miR-124 were JNK3/p38α mitogen-activated protein kinase (MAPK). Overexpression of miR-124 could dramatically inhibit the activation of NF-κB/activating protein-1 (AP-1), the transcription of proinflammatory factors, caspase-9/3, and the cell apoptosis. And opposite results happen when the expression of miR-124 was inhibited. The results suggest that *E. coioides* miR-124 could promote viral replication and negatively regulate host immune response by targeting JNK3/p38α MAPK, which furthers our understanding of virus and host immune interactions.

## Introduction

Viral infections seriously affect the health of organisms including humans. It is important to study the mechanism of viral infection and control the processing of diseases induced by virus. Singapore grouper iridovirus (SGIV) belonging to genus *Ranavirus*, family *Iridoviridae*, is a double-stranded DNA virus with icosahedral symmetry and a diameter of 120–200 nm (1). As a high pathogenic virus of marine fish, SGIV can induce mortality rates of more than 90% and cause major economic losses of the aquaculture (2–6). It is important to clear the SGIV life cycle and the relationship between viral infection and host immunity for controlling the disease induced by SGIV. In recent years, the researchers found that some non-coding RNA could be involved in SGIV infection and replication (7, 8).

MicroRNAs (miRNAs) are small non-coding RNAs with about 22–25 nucleotides in length (9). Since miRNA was found in the embryonic development of *Caenorhabditis elegans*, multiple roles of miRNA were gradually demonstrated (10–12). By binding to the 3′ untranslated region (UTR) of mRNA, miRNA can regulate the translation and expression of genes to affect the processing of proliferation, metabolism, apoptosis, immunity, growth, and plasticity of neurons, etc. (13–15). MiRNAs can also regulate viral replication by adjusting innate immune response or apoptosis of host (16): miR-214 enhances the expression of the target gene AMPK and promotes Snakehead vesiculovirus (SHVV) replication by reducing the expression of type I interferon regulator (17); miR-731 can increase *Cytomegalovirus* replication in the early stage of infection by inhibiting the expression of IRF7 and p53, IFN-I response, cell apoptosis, and cell cycle arrest (18).

In mammals, miR-124 participates in the development and progression of cancer, nervous system, and host–pathogen: miR-124 inhibits cell proliferation in hepatocellular carcinoma by targeting PIK3CA and plays important roles in many cancers *via* the inhibition of PI3K/Akt pathway (19); miR-124 in the brain plays a key role in neurogenesis, neuronal differentiation, and synaptic plasticity in adults (20, 21); and miR-124 has a broad spectrum antiviral activity against influenza A virus (IAV) and respiratory syncytial virus (RSV) (22). To date, whether miR-124 affects the entry of virus remains unknown. In low vertebrate fish, the roles of miR-124 in the interactions of virus and host immune response need to be explored.

*Epinephelus coioides* is an economically mariculture fish in southern China and Southeast Asia, and SGIV can kill large numbers of *E. coioides* in a short time (1–6). Based on this information, the aim of this study was to explore the expression pattern of miR-124 response to SGIV infection; the role of miR-124 in the entry of SGIV, viral titers, and the expressions of viral genes; and immune response of host SGIV-induced cell apoptosis.

## Materials and Methods

### Cells, Virus, and MicroRNAs

The *E. coioides* spleen cells (GS cells, College of Marine Sciences, South China Agricultural University, China) were established in culture medium with 10% fetal bovine serum (Gibco, USA) at 28°C in Leibovitz’s L-15 medium (Gibco, USA). Because of the source, GS cells are good for studying the functions of *E. coioides* miRNAs (8, 23). Because SGIV cannot induce typical apoptosis in GS cells, the fathead minnow (FHM) cells have been verified to be good for analyzing SGIV-induced apoptosis (8, 23, 24). FHM cells herein were used for apoptosis analysis. FHM cells were cultured in Leibovitz’s L-15 medium (Gibco, USA) embodying 10% fetal bovine serum (Gibco, USA) at 28°C (8, 23, 24). SGIV used for viral-infection experiments was originally obtained by our lab as previously described (1). The miRNAs, including control mimics, miR-124 mimics, control inhibitors, and miR-124-specific inhibitors, were purchased from RiboBio (China).

### Total RNA Extraction and cDNA Synthesis

The RNA was isolated from *E. coioides* tissues from the liver, spleen, intestine, trunk kidney, gills, head kidney, skin, muscle, brain, and heart using TRIzol reagent (Invitrogen, Canada) and purified with DNase I (Promega, USA). RNA from the GS cells infected by SGIV was isolated using TRIzol reagent according to the manufacturer’s instruction. The cDNA was synthesized using ReverTra Ace kit (Toyobo, Japan), and the specific miR-124 reverse transcription was obtained using miRNA RT kit (RiboBio, China). The reverse transcription was performed in a final volume of 20 µl containing 4 µl of 5× RT buffer, 1 µl of RT polymerase, 1 µl of miR-124-specific RT primer, and 14 µl of the denatured RNA. The reverse transcription condition was 42°C for 60 min, followed by 70°C for 10 min and 4°C for 15 min. The products obtained were used as a template in quantitative real-time PCR amplification (qPCR).

### Expression Analysis

MiR-124 level was determined using Bulge-Loop™ miRNA qRT-PCR kit (RiboBio, China), and the U6 was used as the reference genes. qPCR was performed with SYBR Green Real-Time PCR Master Mix (Toyobo) at Applied Biosystems QuantStudio 5 Real Time Detection System (Thermo Fisher, USA). The reaction system was 10 µl including 5 µl of SYBR qPCR mix, 0.3 µl of forward and reverse bulge-loop miRNA primer, 3.4 µl of PCR-grade water, and 1 µl of diluted miR-124-specific cDNA. The reaction condition was 95°C for 5 min, followed by 40 cycles of 94°C for 5 s, 56°C for 10 s, and 72°C for 15 s. The expression of the genes was normalized to reference gene and calculated with the 2^−ΔΔCt^ method.

### Western Blotting Analysis

Cells were lysed in pierce IP lysis buffer (Thermo Fisher) and separated by 10% sodium dodecyl sulfate–polyacrylamide gel electrophoresis (SDS-PAGE); and after electrophoresis, the proteins were transferred onto polyvinylidene difluoride (PVDF) membranes (Millipore, USA). In our lab, 5% bovine serum albumin (BSA) diluted rabbit anti-MCP antibody (1:1,000 dilution) was prepared, in which the patent number was CN111363758A (8). P-JNK3(1:1,000 dilution), P-p38 mitogen-activated protein kinase (MAPK) (1:1,000 dilution), caspase-3 (1:1,000 dilution), cleaved caspase-3 (1:1,000 dilution), and rabbit anti-β-tubulin antibody (1:2,000 dilution) was purchased from Abcam. Horseradish peroxidase (HRP)-conjugated goat anti-rabbit (1:5,000) was purchased from KPL (USA). And then we used the HRP-DAB Chromogenic Substrate Kit (Tiangen, China) to visualize according to the manufacturer’s instructions and took photos.

### Confocal Microscopy and Single-Particle Imaging Assay

To explore the effect of miR-124 on the entry of SGIV, we explored the miR-124 on virus entry using confocal imaging. Fluorescence images were observed through ZEISS LSM 7 DUO confocal microscope. The fluorescent label, Alexa Fluor 647 (labelled SGIV), and 4% paraformaldehyde were purchased from Invitrogen. The lipophilic dye, DiO, which indicates the cell boundaries (green), was purchased from Biotium, USA. Fluorescence emission was collected and imaged through a 100× (numerical aperture, 1.4) oil immersion objective. A 488-nm Ar-Kr laser was used to excite DiO signals, and a 500- to 550-nm bandpass filter was used for emission. For quantification analysis, confocal images were obtained by noise filtering, edge detection, and fluorescence signal extraction using a MATLAB program. Approximately 60 cells were randomly analyzed by the MATLAB program to calculate the number of SGIV particles in the cytoplasm.

### The Cytopathic Effect

To evaluate the effects of miR-124 on virus infection, control mimics, miR-124 mimics, control inhibitors, or miR-124 inhibitors (100 nM) were transfected into GS cells in 24-well plates using Lipofectamine RNAiMAX (Invitrogen, USA) according to the manufacturer’s instructions. Twenty-four hours after transfection, the cells were then infected with SGIV for 12-h point infection, and photos of the cell morphology were taken.

### Virus Titer Assay

To detect the effect of miR-124 on SGIV production, the viral titer was evaluated by TCID_50_ analysis. GS cells were transfected with the miR-124 mimics (100 nM) in 24-well plates for 24 h and then infected with SGIV for 24 h. Cells were collected and freeze–thawed three times at −80°C. The cell lysate was then serially diluted and used for GS cell infection in 96-well plates. About 6 days after infection, the viral titer was calculated using TCID_50_ analysis.

### Virus Gene Analysis

To evaluate the effects of miR-124 on virus infection, the miRNAs (100 nM) were transfected into GS cells in 12-well plates. Twenty-four hours after transfection, the cells were infected with SGIV for 24 h, and then the expressions of the viral genes (*MCP*, *VP19*, *ICP18*, and *LITAF*) were detected by RT-qPCR with the β-actin as the reference gene. And the Western blotting analysis was used to detect the protein synthesis of SGIV MCP.

### Plasmid Construction

According to the transcriptome data and the BLAST information, the GenBank accession numbers of JNK3 and p38α MAPK were KT385696.1 and JN408831.1, respectively. The primers JNK3-3′ UTR-F/JNK3-3′ UTR-R, and p38α-3′ UTR-F/p38α-3′ UTR-R (**Table 1**), were designed to amplify JNK3-3′UTR and p38α-3′UTR cDNA. PCR in a final volume of 25 μl was as follows: 1 μl of template DNA, 1 μl of each primer, 12.5 μl of LA Taq polymerase (Takara), and 9.5 μl of H_2_O. The conditions for PCR amplification were as follows: 34 cycles of 94°C, 30 s; 57°C, 30 s; and 72°C, 30 s, followed by 72°C for 5 min. The PCR products were purified and cloned into pmiR-RB-Report™ (RiboBio, China). The restriction sites are *Xho*I and *Not*I. And the recombinant plasmid (pmiR-JNK3 and pmiR-p38α) was confirmed by DNA sequencing. And the primers JNK3-mut-3′UTR-F/JNK3-mut-3′UTR-R and p38α-mut-3′UTR-F/p38α-mut-3′UTR-R were designed to make mutant plasmid (pmiR-mut-JNK3 and pmiR-mut-p38α).

**Table 1:** The primers of this study.

Primer	Sequence (5′–3′)
β-Actin-F	TGCTGTCCCTGTATGCCTCT
β-Actin-R	CCTTGATGTCACGCACGAT
VP19-RT-F	TCCAAGGGAGAAACTGTAAG
VP19-RT-R	GGGGTAAGCGTGAAGACT
LITAF-RT-F	GATGCTGCCGTGTGAACTG
LITAF-RT-R	GCACATCCTTGGTGGTGTTG
MCP-RT-F	GCACGCTTCTCTCACCTTCA
MCP-RT-R	AACGGCAACGGGAGCACTA
ICP18-RT-F	ATCGGATCTACGTGGTTGG
ICP18-RT-R	CCGTCGTCGGTGTCTATTC
JNK3-RT-F	CCAGGACCGCAGGCACCAGTT
JNK3-RT-R	GTGGCGCACCATTTCTCCCATAA
p38α-RT-F	CCTCAACAACATCGTCAAGTG
p38α-RT-R	GGCTTCAAGTCTCTGTGGAT
JNK3-3′UTR-F	CCCTCGAGCCCCCCCTCCTCCTCCATAA
JNK3-3′UTR-R	TTGCGGCCGCCAGGCAGGCGGCTAGTCACC
p38α-3′UTR-F	CCCTCGAGCAGAACCATGACATTCAAGTG
p38α-3′UTR-R	TTGCGGCCGCCAGAGTAACAAAAACAGCAAA
JNK3-mut-3′UTR-F	AATTCTAGGCGATCGCTCGAGCCCCCC
JNK3-mut-3′UTR-R	TTTTATTGCGGCCAGCGGCCGCCCTTACGCGCCATAGTCACCTGCAACAC
p38α-mut-3′UTR-F	AATTCTAGGCGATCGCTCGAGCAGAACCATGAGTAAGTACACGGAGCCGTC
p38α-mut-3′UTR-R	TTTTATTGCGGCCAGCGGCCGCCAG
TNFα-RT-F	GTGTCCTGCTGTTTGCTTGGTA
TNFα-RT-R	CAGTGTCCGACTTGATTAGTGCTT
IL-6-RT-F	CTCTACACTCAACGCGTACATGC
IL-6-RT-R	TCATCTTCAAACTGCTTTTCGTG
IL-8-RT-F	GCCGTCAGTGAAGGGAGTCTAG
IL-8-RT-R	ATCGCAGTGGGAGTTTGCA
IL-1β-RT-F	AACCTCATCATCGCCACACA
IL-1β-RT-R	AGTTGCCTCACAACCGAACAC

### MicroRNA Target Prediction

The RNAhybrid (https://bibiserv.cebitec.uni-bielefeld.de/rnahybrid), TargetScan (http://www.targetscan.org/cgi-bin/), and miRanda (http://www.microrna.org/microrna/) were used to predict the putative targets of miR-124. And the 3′ UTR regions of the target gene of miR-124 of grouper immune-related genes were collected from the National Center for Biotechnology Information (NCBI) database (https://www.ncbi.nlm.nih.gov/).

To analyze the relationship between miR-124 and the gene JNK3/p38α MAPK, GS cells were co-transfected with miR-124 mimics/control mimics (100 nM), luciferase reporter vector (pmiR-JNK3/pmiR-p38α MAPK, 800 ng), and (pmiR-mut-JNK3/pmiR-mut-p38α MAPK, 800 ng) in 24-well plates for 24 h. Luciferase activity was detected using the Dual-Luciferase Reporter Assay system (Promega, USA). The specificity of target was ascertained by the relative luciferase activity of Firefly/Renilla.

### Dual Luciferase Reporter Assays

In order to verify the role of miR-124 in the transcriptional regulation of NF-κB and activating protein-1 (AP-1), control mimics, miR-124 mimics, control inhibitors, or miR-124 inhibitors (100 nM) were co-transfected with 150 ng of NF-κB/AP-1-dependent firefly luciferase reporter plasmid and 40 ng of Renilla luciferase vectors into GS cells at 24-well plates using luciferase reporter assay for 24 h. The cells were infected with SGIV for 12/24 h and harvested using the Dual-Luciferase^®^ Reporter Assay System (Promega, USA) to measure the luciferase activities according to the manufacturer’s instructions.

### Cell Apoptosis Analysis

It was demonstrated that SGIV can induce the typical apoptosis in FHM cells (24). To explore the function of miR-124 in SGIV-induced cell apoptosis, control mimics, miR-124 mimics, control inhibitors, and miR-124 inhibitors at 100 nM were transfected into FHM cells for 24-well plates by three replicates. After 24 h of SGIV infection, the cells were harvested, and the apoptosis was detected by both the terminal deoxy nucleotidyl transferase (TdT)-mediated dUTP nick-end labeling (TUNEL) assay using fluorescence microscope and flow cytometry using the Annexin V-FITC apoptosis detection kit (Beyotime, China) according to the manufacturer’s instructions. Each sample was analyzed in triplicate. Data acquisition and analysis were performed using a flow cytometry system (Beckman Coulter, USA) and FlowJo VX software.

### Caspase-9/3 Activity Analysis

To detect the effect of miR-124 in caspase-9/3 activity, the Caspase Fluorometric assay kit (BioVision, USA) was used to test the activity of caspase-9/3 according to the manufacturer’s instructions. FHM cells at 24-well plates transfected with 100 nM of control mimics, miR-124 mimics, control inhibitors, or miR-124 inhibitors were infected by SGIV for 24 h. The cells were lysed in 50 µl of cold lysis buffer on ice for 10 min and centrifuged at 1,800 rpm for 3 min, and the supernatant was retained. The reaction system was prepared: 50 µl of the supernatant, 50 µl of 2× reaction buffer, 5 µl of caspase-9/3 fluorogenic substrate (LEHD-AFC/DEVD-AFC), and 0.5 µl of fresh DTT. After incubation at 37°C for 1 h, fluorescence was measured (excitation 400 nm, excitation 505 nm) by Thermo Scientific™ Varioskan™ LUX (Thermo Fisher, USA).

### Statistical Analysis

All of the data expressed as mean ± standard error of the mean (SD) were analyzed with GraphPad Prism 7.0 software using one-way ANOVA followed by Duncan’s test. Significance was set at *p* < 0.05.

## Results

### MiR-124 Is Upregulated by Singapore Grouper Iridovirus Infection

*E. coioides* miR-124 was obtained by employing the Solexa deep sequencing approach in our lab (7). As shown in **Table 2**, the sequence was the same except for a difference of 2 or 3 bases in the end of 3′ end in the species, indicating that miR-124 is highly conserved between species.

**Table 2:** Sequence of miR-124 in different species.

MiRNA name	Sequence (5′–3′)	Species	miRBase number
eco-miR-124	UAAGGCACGCGGUGAAUGCCAA	*Epinephelus coioides*	Undetermined
dre-miR-124	UAAGGCACGCGGUGAAUGCCAA	*Danio rerio*	MIMAT0001819
hsa-miR-124-3p	UAAGGCACGCGGUGAAUGCCAA	*Homo sapiens*	MIMAT0000422
bta-miR-124a	UAAGGCACGCGGUGAAUGCCAAG	*Bos taurus*	MIMAT0003811
dme-miR-124-3p	UAAGGCACGCGGUGAAUGCCAAG	*Drosophila melanogaster*	MIMAT0000351
cfa-miR-124	UAAGGCACGCGGUGAAUGCCA	*Canis familiaris*	MIMAT0006657
mmu-miR-124-3p	UAAGGCACGCGGUGAAUGCC	*Mus musculus*	MIMAT0000134
rno-miR-124-3p	UAAGGCACGCGGUGAAUGCC	*Rattus norvegicus*	MIMAT0000828

The red font indicates completely the same sequence.

**Figure 1:**
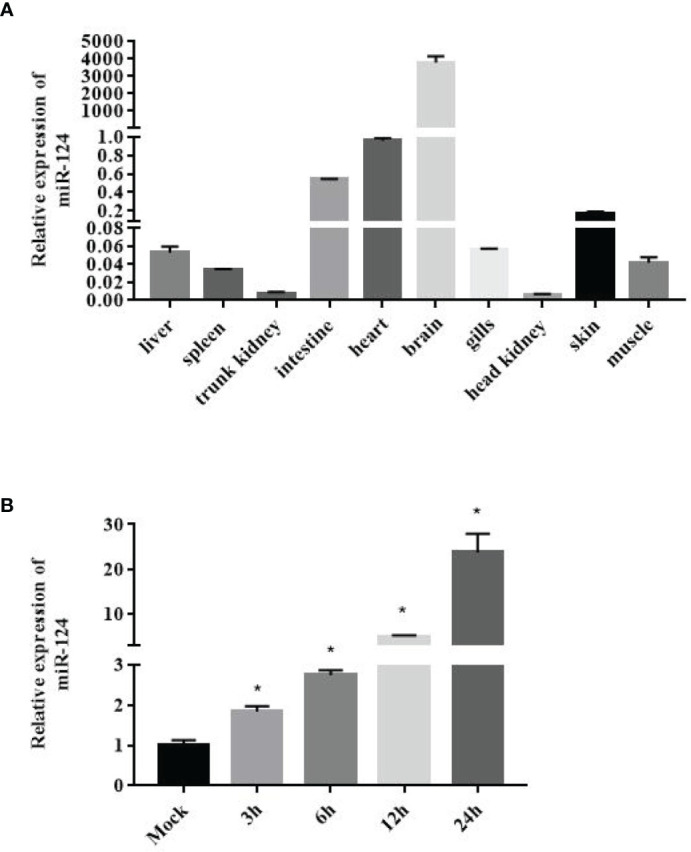
Expression of miR-124 in tissues and in response to Singapore grouper iridovirus (SGIV) infection in the GS cell. **(A)** Expression of miR-124 was detected in the 10 tissues. Data are presented as mean ± SD, N = 3. **(B)** Expression of miR-124 in response to SGIV infection in the GS cell. The GS cells were incubated in 24-well plates and after 24 h were infected by SGIV. U6 was used as internal reference. The control and SGIV infection groups of the miR-124 expression significant differences at each time points are indicated with * (*p* < 0.05). Data are presented as mean ± SD, N = 4.

To obtain the tissue-specific distribution profiles of *E. coioides* miR-124, the total RNA of the 10 tissues from healthy *E. coioides* was extracted, and qPCR was used to quantify the expression of *E. coioides* miR-124. MiR-124 was detected in all of the tissues, and the miR-124 was specifically expressed in the brain, followed by the heart, intestine, skin, liver, gills, muscle, spleen, trunk kidney, and head kidney (**Figure 1A**).

In order to characterize the expression profile of miR-124 after SGIV infection, the expression of miR-124 under the stimulation of SGIV was examined by qPCR. The expression of miR-124 was upregulated during the SGIV infection (**Figure 1B**) (*p* < 0.05).

### MiR-124 Promoted the Singapore Grouper Iridovirus Replication

**Figure 2:**
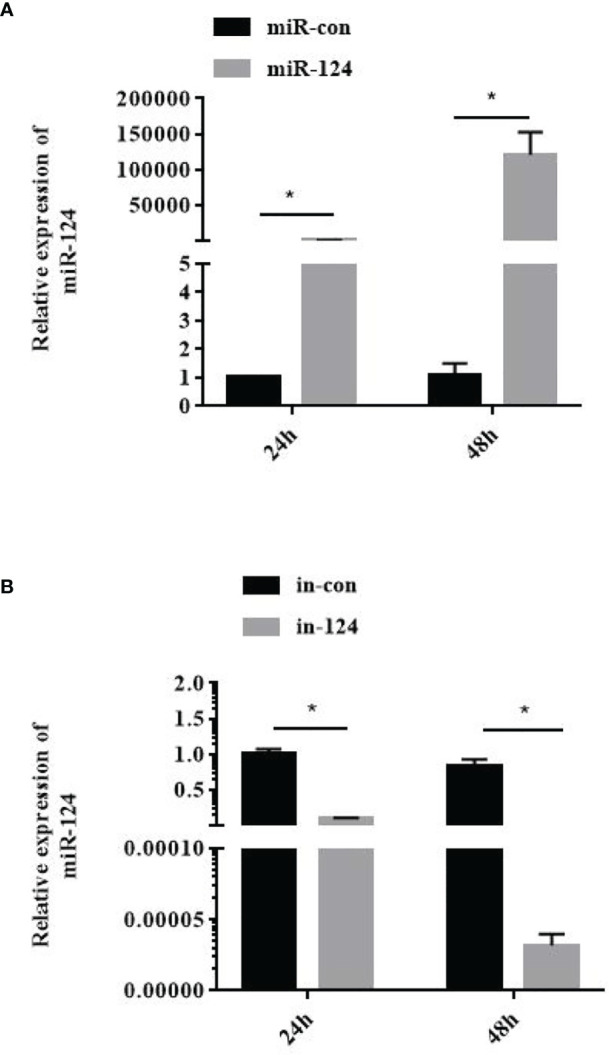
Efficiency detection of miR-124 overexpression. 100nM control mimics **(A)**, miR-124 mimics **(A)**, control inhibitors **(B)**, or miR-124 inhibitors **(B)** were transfected into the GS cells in 12 well plate. The cells at 24 h and 48 h was collected and the expression of miR-124 was detected. U6 was used as internal reference. Control mimics, miR-con; miR-124 mimics, miR-124; control inhibitors, in-con; miR-124 inhibitors, in-124. All data are presented as Mean ± SD, N = 4. Compared with the control group, significant difference of experimental group is indicated with *(*P* < 0.05).

To examine the efficiency of miR-124 mimics or inhibitors, the expression of miR-124 of the GS cells transfected with miR-124 mimics (100 nM) or inhibitors (100 nM) for 24 and 48 h was examined. As shown in **Figure 2A**, the significantly higher expression of miR-124 was detected in the cells with the miR-124 mimics for 48 h (*p* < 0.05), and the miR-124 was significantly downregulated in the cells transfected with the miR-124 inhibitor for 48 h (**Figure 2B**) (*p* < 0.05), showing that it is still efficient at 48 h post transfection with miR-124 mimics or inhibitor.

**Figure 3:**
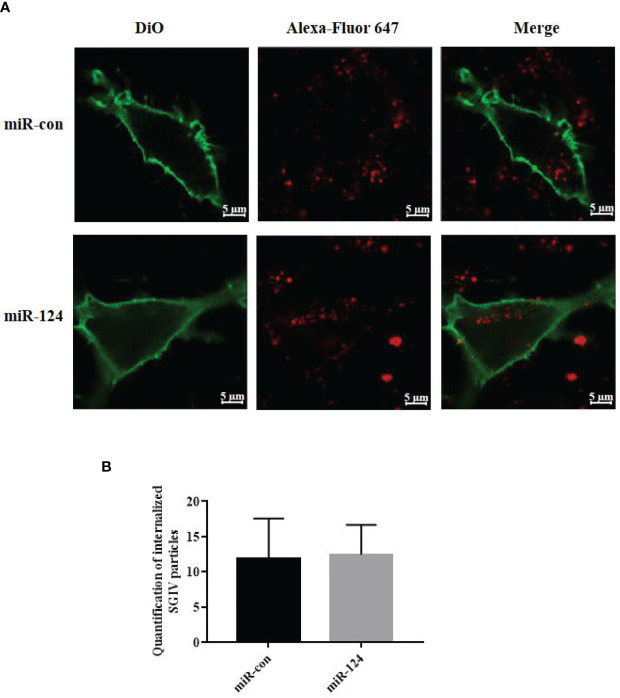
The effect of miR-124 on the entry of Singapore grouper iridovirus (SGIV). **(A)** Three-dimensional confocal images of SGIV attachment in control mimics (miR-con) or miR-124 mimics (miR-124) transfected cells. The samples were stained with DiO to show the cell boundaries (green). The SGIV was labeled Alexa Fluor 647 (red). Scale bars represent 5 μm. **(B)** Quantification of internalized SGIV particles. Over 60 cells were randomly selected and analyzed by MATLAB program. The data are indicated as mean ± SD, N = 60.

To explore the effect of miR-124 on the entry of SGIV, virus entry in the cells transfected with miR-124 mimics was analyzed using confocal imaging. Compared with the cells transfected with the control mimics, cells transfected with miR-124 mimics were not significantly different on SGIV entry (**Figures 3A, B**), suggesting that miR-124 might not be involved in the entry of SGIV.

**Figure 4:**
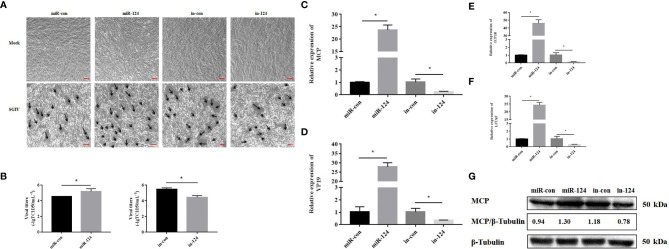
Effect of miR-124 on virus infection and replication. **(A)** The cytopathic effect (CPE) of Singapore grouper iridovirus (SGIV)-infected miRNA-transfected GS cells, and the black arrows indicate the severity of CPE caused by SGIV infection at 24 h.The scale is 50 μm. **(B)** The viral titers in each group were measured using the TCID_50_ method. Data are presented as mean ± SD, N = 3. **(C–F)** The expression levels of SGIV genes (*MCP*, *VP19*, *ICP18*, and *LITAF*). Data are presented as mean ± SD, N = 4. Compared with the control group, significant difference of experimental group is indicated with *(*P* < 0.05). **(G)** The level of SGIV MCP proteins was detected by Western blotting, the band intensity was calculated, and ratios of target protein/β-tubulin were assessed by ImageJ software. Control mimics, miR-con; miR-124 mimics, miR-124; control inhibitors, in-con; miR-124 inhibitors, in-124.

The GS cells transfected with miR-124 mimics were infected with SGIV for 24 h. Subsequently, the SGIV-induced cytopathic effects (CPEs), the SGIV production, and expression of the viral genes were analyzed. And the CPE was increased in the cells transfected with miR-124 mimics and decreased in the cells transfected with miR-124 inhibitor (**Figure 4A**). The viral titer, which was used to evaluate viral production, of the miR-124 overexpression cells was significantly higher than that of the control cells, while that of the miR-124 downregulated cells was significantly lower than that of the control cells (**Figure 4B**) (*p* < 0.05). Furthermore, the transcription levels of SGIV genes (*MCP*, *VP19*, *ICP18*, and *LITAF*) were examined. The expression of viral genes *MCP*, *VP19*, *ICP18*, and *LITAF* were significantly upregulated in the cells transfected with miR-124 mimics, which were downregulated in the cells transfected with miR-124 inhibitor (**Figures 4C–F**). By Western blotting, the protein level of MCP in the cells transfected with miR-124 mimics (1.30) was higher than that of the control group (0.94), while the protein level of MCP in the cells transfected with miR-124 inhibitor (0.78) was lower than that of the control group (1.18) (**Figure 4G**), showing that overexpression of miR-124 could enhance the protein synthesis of SGIV MCP and that inhibited miR-124 could decrease the synthesis of MCP.

### MiR-124 Regulates Innate Immune Response of *Epinephelus coioides*

**Figure 5:**
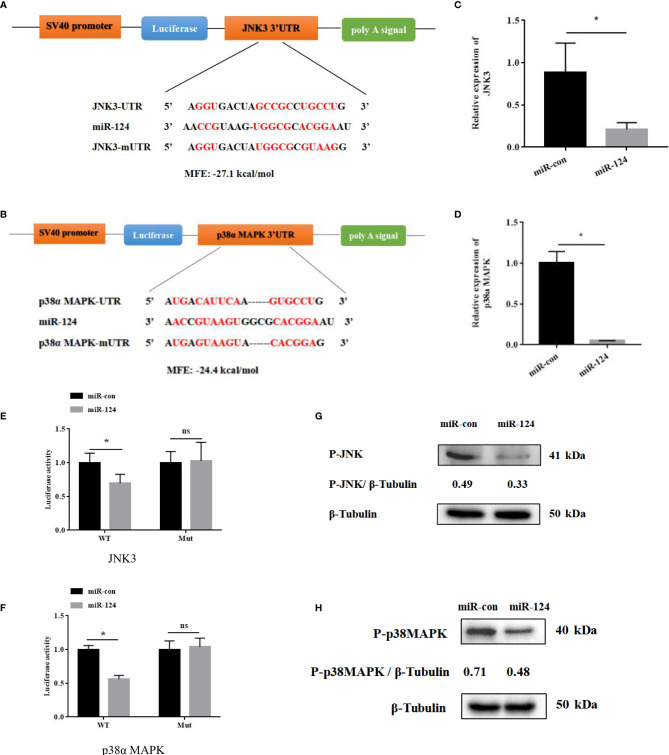
JNK3 and p38α MAPK were identified as the targets of miR-124. **(A)** Diagram of JNK3 3′ UTR-containing reporter constructs. **(B)** Diagram of p38α 3′ UTR-containing reporter constructs. **(C)** The mRNA levels of JNK3 were reduced in GS cells treated with miR-124 mimics for 24 h. Actin was used as internal reference. **(D)** The mRNA levels of p38α were reduced in GS cells treated with miR-124 mimics for 24 h. β-Actin was used as internal reference. **(E)** The 3′ UTR reporter assay was performed in GS cells 24 h after transfection. **(F)** The 3′ UTR reporter assay was performed in GS cells 24 h after transfection. **(G)** The level of P-JNK3 proteins was detected by Western blotting, the band intensity was calculated, and ratios of target protein/β-tubulin were assessed by ImageJ software. **(H)** The level of P-p38 MAPK proteins was detected by Western blotting, the band intensity was calculated, and ratios of target protein/β-tubulin were assessed by ImageJ software. Control mimics, miR-con; miR-124 mimics, miR-124; control inhibitors, in-con; miR-124 inhibitors, in-124. All data are presented as mean ± SD, N = 4. Compared with the control group, significant difference of experimental group is indicated with *(*P* < 0.05), and no significant difference is indicated with ns (*P* > 0.05).

By bioinformatics tools, JNK3 and p38α MAPK were the putative target genes of miR-124, and the binding energy value was −27.1 and −24.4 kcal/mol, respectively. The miR-124 sequence contains a conserved sequence matching the JNK3/p38α MAPK binding 3′-UTR (**Figures 5A, B**). To study the role miR-124 on JNK3 and p38α MAPK, the JNK3 and p38α MAPK mRNAs were examined in the overexpression miR-124 cells by qPCR. The expressions of both JNK3 and p38α MAPK in the cells transfected with miR-124 mimics were significantly downregulated than those in the control groups (**Figures 5C, D**) (*p* < 0.05). To verify the relationship of JNK3/p38α MAPK and miR-124, JNK3/p38α MAPK 3′-UTR or JNK3/p38α MAPK mutant 3′-UTR was cloned into a luciferase reporter vector pmiR-RB-Report. The GS cells transfected with the luciferase reporter containing miR-124 mimics and JNK3/p38αMAPK wild 3′-UTR or JNK3/p38α MAPK mutant 3′-UTR were analyzed. The luciferase activities were significantly reduced in the cells containing JNK3/p38α MAPK wild 3′-UTR (**Figures 5E, F**) (*p* < 0.05), and there was no significant change in the cells of JNK3/p38α MAPK mutant 3′-UTR (*p* < 0.05). And the protein level of P-JNK3/P-p38α MAPK in the cells transfected with miR-124 mimics (0.33 and 0.48) was lower than that of the control group (0.49 and 0.71) (**Figures 5G, H**).

**Figure 6:**
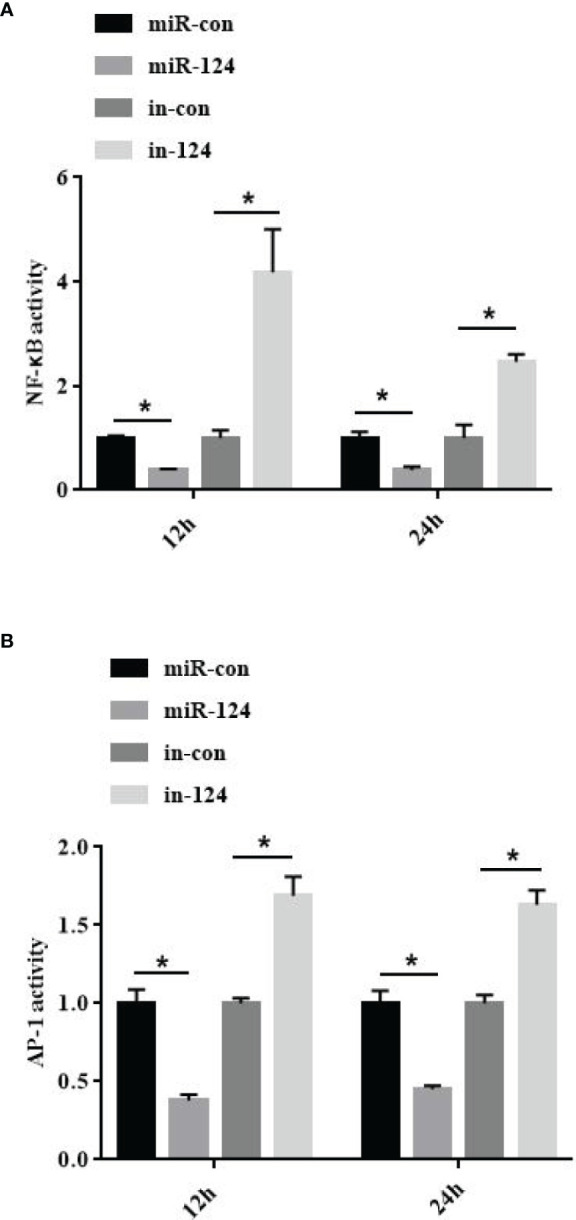
MiR-124 inhibited NF-κB and activating protein-1 (AP-1) activation. The activities of NF-κB **(A)** and AP-1 **(B)** were analyzed by dual luciferase reporter assays. The miRNAs were transfected into the fathead minnow (FHM) cells in 24-well plates. After 24-h transfection, cells were infected with Singapore grouper iridovirus (SGIV) for 12 and 24 h. Relative luciferase activity is measured as a ratio of firefly luciferase activity to Renilla luciferase activity. Control mimics, miR-con; miR-124 mimics, miR-124; control inhibitors, in-con; miR-124 inhibitors, in-124. All data are presented as mean ± SD, N = 4. Compared with the control group, significant difference of experimental group is indicated with *(*P* < 0.05).

To study the effect of miR-124 on the transcriptional activity of NF-κB and AP-1, the control mimics, miR-124 mimics, control inhibitors, and miR-124 inhibitors at 100 nM were transfected into GS cells for 24 h; and then the cells were infected with SGIV and collected at 12 and 24 h post infection. The activations of the NF-κB and AP-1 were significantly reduced in the cells transfected with miR-124 mimics (*p* < 0.05), and those of the NF-κB and AP-1 were significantly upregulated in the cells transfected with miR-124 inhibitor compared with the control group (*p* < 0.05) (**Figures 6A, B**).

**Figure 7:**
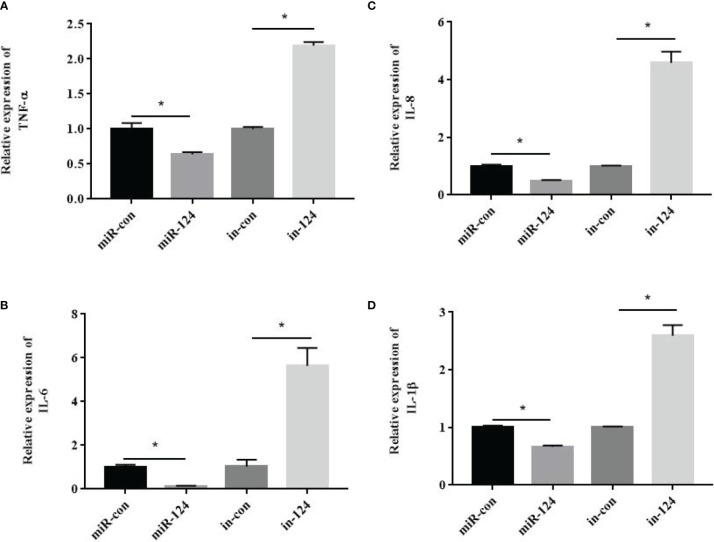
The effect of miR-124 on the transcription of proinflammatory factors. β-Actin was used as internal reference. The transcription of TNF-α **(A)**, IL-6 **(B)**, IL-8 **(C)** and IL-1β **(D)** were analyzed by qRT-PCR. control mimics: miR-con; miR-124 mimics: miR-124; control inhibitors: in-con; miR-124 inhibitors: in-124. All data are presented as Mean ± SD, N = 4. Compared with the control group, significant difference of experimental group is indicated with *(*P* < 0.05).

To verify the roles of miR-124 in innate immunity, GS cells transfected with control mimics/miR-124 mimics/control inhibitors/miR-124 inhibitors were infected with SGIV, and the expressions of the proinflammatory factors TNF-α, IL-6, IL-8, and IL-1β were detected. The expressions of these factors in the cells transfected with miR-124 mimics were significantly lower than those in the control group; those of the miR-124 inhibitors were significantly higher those of the control inhibitor (**Figure 7**) (*p* < 0.05), suggesting that miR-124 significantly reduced the transcription levels of the *E. coioides* immune-related factors.

### MiR-124 Inhibited the Singapore Grouper Iridovirus-Induced Cell Apoptosis

SGIV infection can cause cell apoptosis in FHM cells (8). To explore the influence of miR-124 on SGIV-induced cell apoptosis, miR-124 mimics/control mimics/control inhibitors/miR-124 inhibitors were transfected into FHM cells, and the cells were infected by SGIV for 24 h. Subsequently, the cells were harvested, and the apoptosis was analyzed.

**Figure 8:**
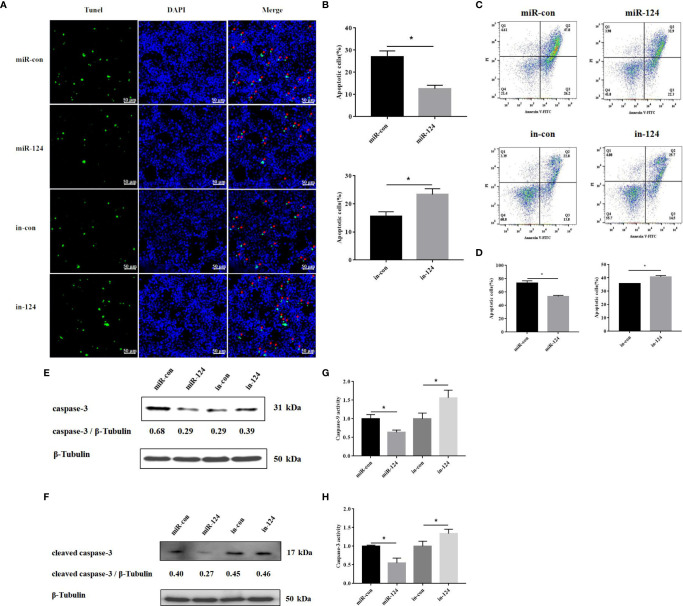
The effect of miR-124 on apoptosis of fathead minnow (FHM) cells. **(A)** Detection of apoptosis by TUNEL assay. The green fluorescent markers indicate fractured DNA fragments, and the blue fluorescent markers represent the nucleus. The red arrows indicate the fractured DNA fragments. Scale bars represent 50 μm. **(B)** Comparison of apoptosis rates of FHM cells in different groups. **(C)** Singapore grouper iridovirus (SGIV)-induced apoptotic bodies in FHM cells. Q1: PI+, Annexin−. Q2: PI+, Annexin+. Q3: PI−, Annexin+. Q4: PI−, Annexin−. Q2 and Q3 represent the percentage of late apoptosis and early apoptosis, respectively. **(D)** The graph is based on the average of three parallel experimental data. **(E, F)** The level of caspase-3 and cleaved caspase-3 proteins was detected by Western blotting, the band intensity was calculated, and ratios of target protein/β-tubulin were assessed by ImageJ software. **(G, H)** The activity of caspase-9 and caspase-3 after SGIV infection. Control mimics, miR-con; miR-124 mimics, miR-124; control inhibitors, in-con; miR-124 inhibitors, in-124. All data are presented as mean ± SD, N = 3. Compared with the control group, significant difference of experimental group is indicated with *(*P* < 0.05).

By TUNEL assay, the fractured DNA was labeled using fluorescein isothiocyanate (FITC)-conjugated UTP. The apoptotic cells were counted with florescence microscope. As shown in **Figure 8A**, the fractured DNA fragments were decreased in the cells transfected with miR-124 mimics and increased in the cells transfected with miR-124 inhibitor. The apoptosis rates in the cells transfected with control mimics and miR-124 mimics were 27.0% and 12.7%, respectively (**Figure 8B**). The apoptosis rates were 15.7% and 23.3% in the cells transfected with miR-124 inhibitor (**Figure 8B**).

Similar results occurred in the analysis of flow cytometry. By flow cytometry, the apoptosis rate (sum of early apoptosis and late apoptosis) in the cells transfected with control mimics or miR-124 mimics was 74.0% or 55.2%, respectively (**Figures 8C, D**). The apoptosis rate (sum of early apoptosis and late apoptosis) in control cells was 35.8% and 40.2% in the cells transfected with miR-124 inhibitor (**Figures 8C, D**).

By Western blotting, the protein level of caspase-3 in the cells transfected with miR-124 mimics (0.29) was lower than that of the control group (0.68), while the protein level of caspase-3 in the cells transfected with miR-124 inhibitor (0.39) was higher than that of the control group (0.29) (**Figure 8E**); the protein level of cleaved caspase-3 in the cells transfected with miR-124 mimics (0.27) was lower than that of the control group (0.40), while the protein level of cleaved caspase-3 in the cells transfected with miR-124 inhibitor (0.46) was higher than that of the control group (0.45) (**Figure 8F**), showing that overexpression of miR-124 could decrease the protein of caspase-3 and cleaved caspase-3 and that inhibited miR-124 could enhance the protein of caspase-3 and cleaved caspase-3. The activity of caspase-9 and caspase-3 in the cells transfected with miR-124 mimics was significantly inhibited, as compared with that in the control group (**Figures 8G, H**) (*p* < 0.05). The results indicated that miR-124 significantly suppressed SGIV-induced cell apoptosis.

## Discussion

It is well known viral infections seriously affect the health of organisms. To date, non-coding RNA as a key factor has been involved in multiple biological processing including its roles in the response to pathogen stimulates. Non-coding RNA and miRNA regulated many functions of cells, including proliferation, metabolism, apoptosis, immunity, growth, and plasticity of neurons (13–15). However, the relationship of miRNA such as miR-124 and the viral entry remains unknown. In this study, the roles of marine fish *E. coioides* miR-124 in the infection and replication of SGIV and the innate immune response of host were explored.

Except for the difference of 2 or 3 bases in the 3′ end, *E. coioides* miR-124 shares the same sequences with other species from worm to humans, suggesting that its roles in these species might be similar (25, 26). In mammals, miR-124 is closely related to brain development, function, and homeostasis (27–29). However, the brain controls the spleen to regulate the function of the immune system from top to bottom; a special brain–spleen connection was recognized, enhancing humoral response autonomously and displaying immune stimulation through physical behavior, which revealed the brain’s control of adaptive immunity, and proposes the possibility of improving immune ability through behavioral intervention (30). Similarly, miR-124 here was specifically expressed in *E. coioides* brain.

The pathogenesis of virus is a complex process, which is the dynamic interaction of many host factors, such as miRNAs as the key factors. Viral infection can affect the expression of host miRNA. *Litopenaeus vannamei* miRNAs were differentially expressed after white spot syndrome virus (WSSV) infection, and the different expression of miRNAs could affect host signaling pathway of immune response (31). SGIV is an important virus of marine cultured fish causing huge financial losses, and SGIV infection can cause a series of host immune responses, including the changes of host miRNA expression (23). Previous studies in our lab have shown that the numbers of miRNA of *E. coioides* occurred with different expression responses to SGIV infection, and the expressions of some miRNAs were increased (7). In this study, miR-124 is upregulated during SGIV infection, indicating that miR-124 would be involved in SGIV infection and replication.

MiRNA could regulate viral infection (32). During viral infection, differential expression profiles of host miRNAs may lead to viral replication with feedback or feedforward effects (33). MiR-214 can directly interact with hepatitis E virus (HEV) RNA to enhance the replication and the genome translation of HEV, and the increase of HEV ORF2 level can upregulate the expression of miR-214 (34). MiR-1307 inhibits the replication of foot and mouth disease virus (FMDV) by the degradation of FMDV VP3 through proteasome pathway (35). In this study, SGIV infection can increase the expression of miR-124, and the upregulated miR-124 could not be involved in SGIV entry, but it could increase SGIV-induced CPE, and virus titers, transcription levels of SGIV genes, and the protein level of SGIV MCP, suggesting that miR-124 can be activated by SGIV infection, and the activated miR-124 could promote the replication of SGIV.

To further explore the roles of miR-124 in innate immune response of *E. coioides*, bioinformatics, gene mutants, Western blotting, etc., were used. By miRNA target prediction and the binding sties mutation of targeting genes, JNK3 and p38α MAPK were important target genes of miR-124: the 3′-UTR of JNK3/p38α MAPK contained the matched binding sequence with miR-124; miR-124 can downregulate the mRNA and protein JNK3/p38α MAPK, and JNK3/p38α MAPK 3′-UTR-contained reporter. As members of MAPK family, JNK3 and p38α MAPK can be activated; can convert the extracellular signals into intracellular signals by stress, viral infection, inflammatory cytokines, and mitotic factors (36–39); and can regulate the cell apoptosis and the viral replication (40–42). In mammals, miR-124 regulates the expression of p38α MAPK to participate in the neurons of signal transduction to translation machinery (43), and miR-124 regulates JNK to induce cell death in CD133^+^ HCC cells (44). Previous studies in our lab demonstrated that SGIV infection can induce the inflammation, and the immune response of host, causing the differential expression of host mRNA such as JNK and p38α MAPK (5, 45). JNK and p38α MAPK were the target genes of miR-124; the high expression of JNK and p38 MAPK would stimulate miR-124; and the expression of miR-124 was upregulated, suggesting that miR-124 could regulate SGIV infection by targeting JNK3 and p38α MAPK.

JNK and p38 MAPK could affect the activity of NF-κB and other transcription factors like AP-1 (3, 5, 8, 45). NF-κB plays key roles in response to various stress stimuli and is the main regulator and initiator of inflammatory response (46–48). Together with AP-1, the activated NF-κB participated in many cellular processes, including apoptosis, proliferation, and differentiation (49). The factors of TNF-α, IL-1β, IL-6, and IL-8 are in synergy with cytokines, which are messengers of the inflammatory cascade (50, 51). In this study, the activation of NF-κB and AP-1 and the expressions of the factors (TNF-α, IL-6, IL-8, and IL-1β) were significantly inhibited in miR-124-overexpressing cells with SGIV infection, suggesting that miR-124 inhibits the activity of NF-κB and AP-1, and the expressions of the inflammatory factors to regulate the innate immunity.

Apoptosis is an innate cellular response to inflammatory response, transmission, and expression of virus. Virus has acquired the ability to regulate host cell apoptosis, control inflammatory response, and escape immune response (52). The Giant seaperch iridovirus (GSIV) ST kinase can activate caspase-9 and caspase-3 and induce the apoptosis (53). SGIV can induce typical apoptosis and increase the activities of caspase-9 and caspase-3 in FHM cells (2, 3, 5, 8, 24). Both grouper miR-146a and miR-122 can reduce the SGIV-induced cell apoptosis and the activation of caspase-9 and caspase-3 (8, 23). To explore the roles of miR-124 in SGIV-induced apoptosis, apoptosis rates and the activation of caspase-9/3 were analyzed. A significant decrease of the SGIV-induced apoptosis was observed in the cells with overexpression miR-124; the protein levels of caspase-3 and cleaved caspase-3 were reduced; and the activity of caspase-9/3 was significantly inhibited in miR-124-overexpressing cells. It has been demonstrated that p38 MAPK mediates the activity of caspase-8/9/3 in human umbilical vein endothelial cells when apoptosis is induced by PEDF; JNK was independently involved in the upregulation of caspase-3 activity; and p38 MAPK was known to act as an upstream regulator of caspase-3 in apoptotic endothelial cells (54–56). Since JNK and p38 MAPK can active caspase-9/3 and induce the cell apoptosis, and miR-124 here can deregulate the expression of JNK and p38 MAPK by targeting 3′-UTR, we speculate that miR-124 might regulate the cell apoptosis and the activities of caspase-9 and caspase-3 *via* JNK and p38 MAPK.

In conclusion, in low vertebrates *E. coioides*, the roles of miR-124 in SGIV infection and replication, and innate immune response were analyzed in this study. The expression level of miR-124 was significantly upregulated during SGIV infection. Overexpression of miR-124 cannot affect SGIV entry but significantly increased SGIV-induced CPE, viral replication, and the expressions of SGIV key genes. MiR-124 can inhibit the activities of NF-κB and AP-1, the expressions of inflammatory factors (TNF-α, IL-6, IL-8, and IL-1β), and the SGIV-induced apoptosis by targeting 3′ UTR of JNK3 and p38α MAPK. This study demonstrated that miR-124 was not related to SGIV entry but promotes SGIV replication and negatively regulates the innate immunity, which provides new insights into understanding the roles of fish miRNAs in virus pathogenesis.

## Data Availability Statement

The datasets presented in this study can be found in online repositories. The names of the repository/repositories and accession number(s) can be found in the article/supplementary material.

## Ethics Statement

The animal study was reviewed and approved by South China Agricultural University. Written informed consent was obtained from the owners for the participation of their animals in this study.

## Author Contributions

Q-WQ and H-YS conceived the experiments and reviewed the drafts of the manuscript. S-WW and L-QW contributed the materials/analysis tools. P-HL wrote the manuscript, conducted the experiment, and analyzed the data. All authors contributed to the article and approved the submitted version.

## Funding

This work was funded by National Natural Science Foundation of China (U20A20102, 31930115), Innovation Group Project of Southern Marine Science and Engineering Guangdong Laboratory (Zhuhai) (311021006), the China Agriculture Research System of MOF and MARA (CARS-47-G16), Marine Fisheries Bureau Key funds and marine projects (GDME-2018C002), the National Key R&D Program of China (2018YFD0900501 and 2018YFC0311302).

## Conflict of Interest

The authors declare that the research was conducted in the absence of any commercial or financial relationships that could be construed as a potential conflict of interest.

## Publisher’s Note

All claims expressed in this article are solely those of the authors and do not necessarily represent those of their affiliated organizations, or those of the publisher, the editors and the reviewers. Any product that may be evaluated in this article, or claim that may be made by its manufacturer, is not guaranteed or endorsed by the publisher.
